# Indole-Based Small Molecules as Potential Therapeutic Agents for the Treatment of Fibrosis

**DOI:** 10.3389/fphar.2022.845892

**Published:** 2022-02-16

**Authors:** Rui Qin, Qian Zhao, Bo Han, Hong-Ping Zhu, Cheng Peng, Gu Zhan, Wei Huang

**Affiliations:** ^1^ State Key Laboratory of Southwestern Chinese Medicine Resources, Hospital of Chengdu University of Traditional Chinese Medicine, School of Pharmacy, Chengdu University of Traditional Chinese Medicine, Chengdu, China; ^2^ Antibiotics Research and Re-Evaluation Key Laboratory of Sichuan Province, Sichuan Industrial Institute of Antibiotics, Chengdu University, Chengdu, China

**Keywords:** indole alkaloids, organ fibrosis, mechanisms, TGF-β/Smad pathway, extracellular matrix

## Abstract

Indole alkaloids are widely distributed in nature and have been particularly studied because of their diverse biological activities, such as anti-inflammatory, anti-tumor, anti-bacterial, and anti-oxidant activities. Many kinds of indole alkaloids have been applied to clinical practice, proving that indole alkaloids are beneficial scaffolds and occupy a crucial position in the development of novel agents. Fibrosis is an end-stage pathological condition of most chronic inflammatory diseases and is characterized by excessive deposition of fibrous connective tissue components, ultimately resulting in organ dysfunction and even failure with significant morbidity and mortality. Indole alkaloids and indole derivatives can alleviate pulmonary, myocardial, renal, liver, and islet fibrosis through the suppression of inflammatory response, oxidative stress, TGF-β/Smad pathway, and other signaling pathways. Natural indole alkaloids, such as isorhynchophylline, evodiamine, conophylline, indirubin, rutaecarpine, yohimbine, and vincristine, are reportedly effective in organ fibrosis treatment. In brief, indole alkaloids with a wide range of pharmacological bioactivities are important candidate drugs for organ fibrosis treatment. The present review discusses the potential of natural indole alkaloids, semi-synthetic indole alkaloids, synthetic indole derivatives, and indole-contained metabolites in organ fibrosis treatment.

**GRAPHICAL ABSTRACT:**
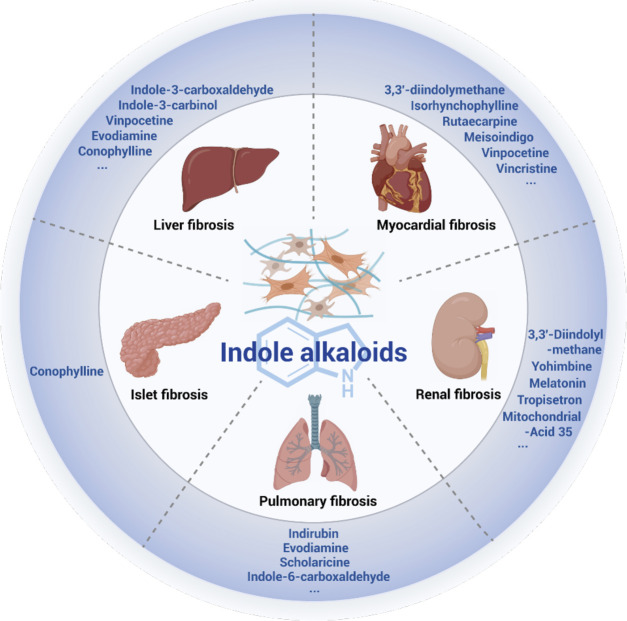


## Introduction

**FIGURE 1:**
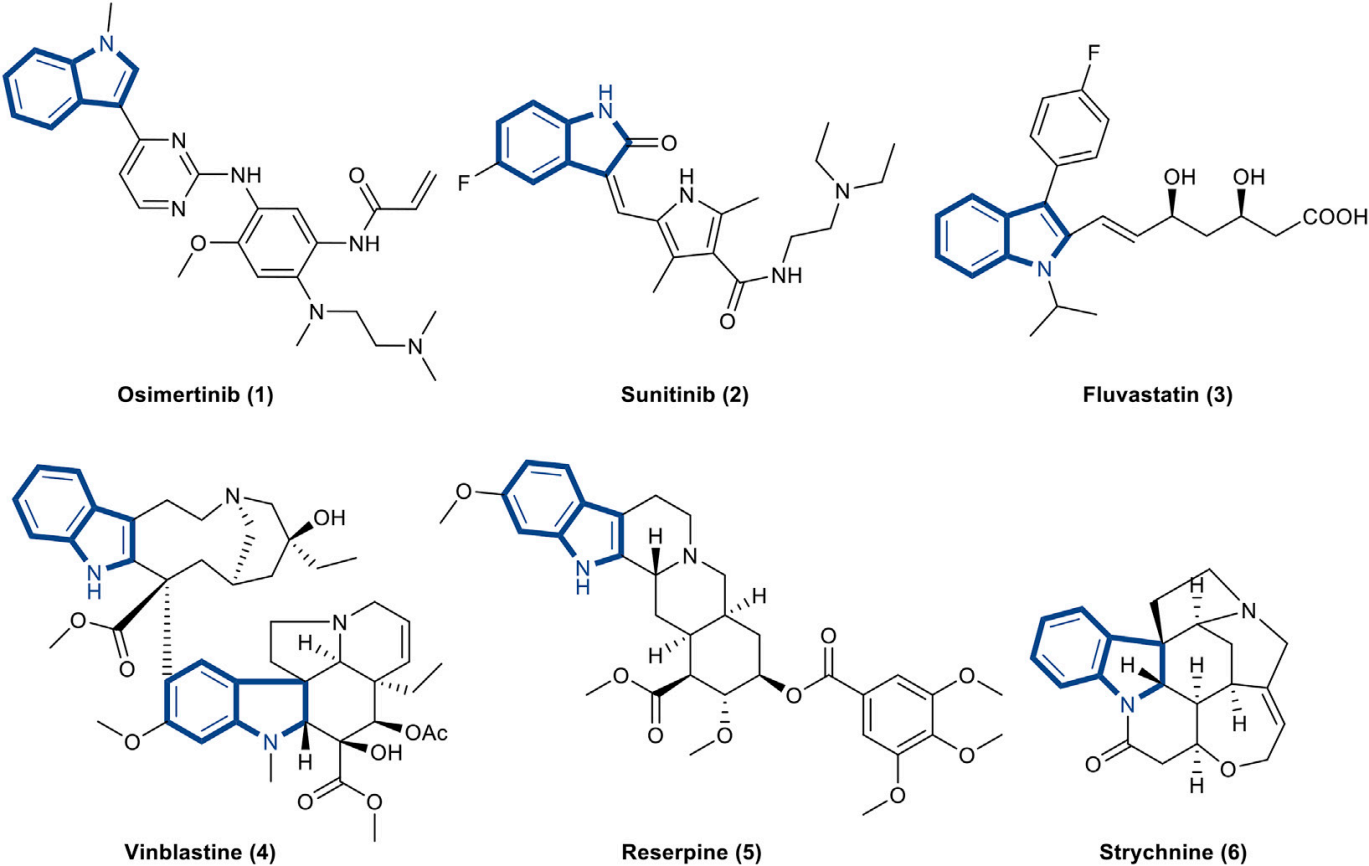
Representative drugs containing indole skeleton. **(1)**. Osimertinib is the first and only NSCLC drug approved for the EGFR T790M mutation (Soria et al., 2018). **(2)**. Sunitinib is a new multi-targeted tyrosine kinase inhibitor for the treatment of tumors (Beuselinck et al., 2015). **(3)**. Fluvastatin is a hydroxymethylglutaryl-CoA (HMG-CoA) reductase inhibitor and a fully synthetic blood lipid lowering drug (Henriksbo et al., 2014). **(4)**. Vinblastine is an antitumor drug extracted from *Catharanthus roseus* that interferes with protein synthesis (Sasaki et al., 2010). **(5)**. Reserpine is an antihypertensive drug found in the roots of *Rauwolfia serpentine* (Abdelfatah and Efferth, 2015). **(6)**. Strychnine is an indole alkaloid extracted from *Strychnos* that excites the spinal cord and enhances skeletal muscle tone Mori et al., 2003.

Indole alkaloids are bicyclic nitrogenous compounds formed by the combination of six-membered benzene and five-membered pyrrole. Such alkaloids are widely distributed in the plant families. They mainly exist in Leguminosae, Loganiaceae, Apocynaceae, Clavicipitaceae, and Rubiaceae (Martins and Nunez, 2015; Zhu et al., 2015). They are the active ingredients of many medicinal plants and have diverse biological activities, such as anti-tumor, anti-inflammatory, and antibacterial, which play important roles in our lives (Ishikawa et al., 2009; Keglevich et al., 2012; Sravanthi and Manju, 2016; Chadha and Silakari, 2017; Singh and Singh, 2018). Well-known natural and synthetic drugs characterized by indole frameworks are very common (Figure 1; Mori et al., 2003; Sasaki et al., 2010; Henriksbo et al., 2014; Abdelfatah and Efferth, 2015; Beuselinck et al., 2015; Soria et al., 2018). The structural modification of these active molecules to screen compounds with novel structure and higher activity is among the hot spots in pharmaceutical chemistry research. Recently, a series of natural, semi-synthetic, synthetic compounds and metabolites that characterize the indole portion of their structures have been used to explore the functional role of indole alkaloids against organ fibrosis, many of which have shown promising outcomes. Indole alkaloids have powerful therapeutic effects on various fibrotic diseases. However, their mechanisms of action are complex and may be related to many signaling pathways in the fibrosis process.

Fibrosis is a highly dynamic process and results from the abnormal regulation of the tissue repair response following multiple types of tissue injury, especially during chronic inflammatory diseases (Wynn and Ramalingam, 2012; Henderson et al., 2020). Fibrosis formation is defined as excessive deposition of extracellular matrix (ECM) components such as collagen and fibronectin, which can affect nearly every organ system, including skin, heart, liver, lung, kidney, and pancreas. In case of sustained or severe injury, ECM components continue to accumulate, leading to structural destruction and dysfunction of organs, and even failure (Kendall and Feghali-Bostwick, 2014; Weiskirchen et al., 2019). In the COVID-19 outbreak at the end of 2019, most of the infected patients developed the sequelae of pulmonary fibrosis, which is one of the health issues we are most concerned about (George et al., 2020). In a study on the inhibition of fibrosis, it was found that although the pathogenesis of fibrosis in different organs is complex and different, the basic common process involves inflammatory stimulation and organ parenchymal cell necrosis, which stimulate the activation of macrophages and monocytes, as well as excessive production of cytokines and chemokines (including TGF-β, CYGF, PDGF, IL-1β, IL-6, IL-11, and TNF-α), ultimately leading to fibrosis (Rockey et al., 2015; Horowitz and Thannickal, 2019). In addition, some non-peptide mediators, such as reactive oxygen species (ROS) and lipid mediators, can also induce fibrogenesis (Sanchez-Valle et al., 2012).

**FIGURE 2:**
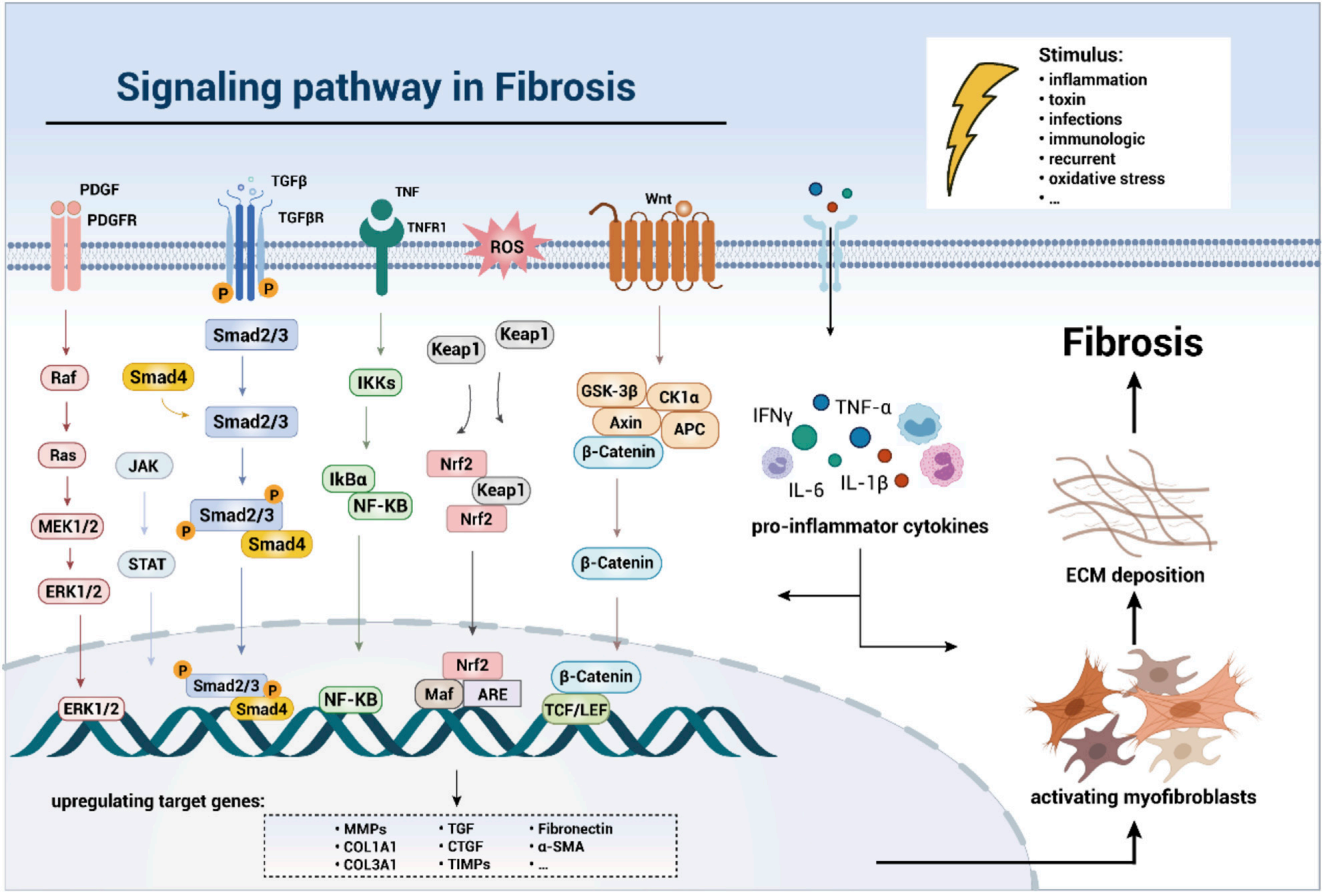
Schematic diagram of the interaction of profibrotic signaling pathway.

Excessive studies had shown that TGF-β1/Smad pathway was an important pathogenic mechanism in organ fibrosis. Smads proteins are downstream signal transducers of TGF-β. TGF-β activates Smad2 and Smad3 after binding to its receptor and then forms a complex with Smad4 to enter the nucleus and bind to target genes to cause ECM production (Chen et al., 2018; Zhang et al., 2020a). Besides, activation of the nuclear factor kappa B (NF-κB) pathway can increase the expression of various pro-inflammatory factors and thus mediate inflammatory activity. NF-κB is a heterodimer composed of two subunits p50 and p65. When stimulated, IκB is phosphorylated and dissociated from NF-κB dimer in the presence of protein kinases and phosphatases, revealing nuclear localization signal of p50 protein, thereby activating NF-κB. Released NF-κB translocates to the nucleus, where it binds to specific IκB sequence and triggers the expression of TNF-α, IL-6, IL-17, and other pro-inflammatory mediators (Yu et al., 2020; Mirzaei et al., 2021). Sustained activation of Wnt/β-catenin pathway is associated with the pathogenesis of fibrotic disorders. When the Wnt pathway is activated, the degradation of free β-catenin in cytoplasm is inhibited and the content of β-catenin is increased. β-catenin enters the nucleus and polymerizes with T cell factors and lymphocyte enhancer factors (TCF/LEF) to form intranuclear complexes, which regulate transcription of target genes and ultimately accelerate the progression of fibrosis (Wang et al., 2018; Schunk et al., 2021). In addition to the above-mentioned pathways, Raf/MEK/ERK, JAK/STAT and other pathways are also involved in the regulation of fibrosis (Figure 2; Guo et al., 2018; Foglia et al., 2019; Liu et al., 2019a; Pompili et al., 2019; Montero et al., 2021). Fibrous diseases reportedly cause up to 45% of deaths in the western developed country (Oruqaj et al., 2015). To date, few drugs have been approved to treat fibrosis, and these drugs can only provide relief and cannot fundamentally reverse or cure the disease. Therefore, it is necessary to further study the pathogenesis of fibrosis and find ideal pharmaceuticals to control it.

Indole is a privileged scaffold in anti-fibrotic drug discovery. Recently, due to the high biological activity of indole alkaloids, many studies have been conducted to explore its anti-fibrosis effect. Indole-based small molecules can improve pulmonary, liver, myocardial, renal, and islet fibrosis by regulating the NF-κB pathway, Wnt/β-catenin cascade, TGF-β/Smad pathway, Nrf-2/HO-1 cascade, PTEN/AKT signaling pathway, and so on. The use of indole alkaloids for the treatment of organ fibrosis is promising. The aim of this review is to verify the therapeutic effects and activities of indole alkaloids and its derivatives on organ fibrosis.

### Pulmonary Fibrosis

Pulmonary fibrosis is a chronic interstitial lung disease caused by a variety of internal and external pathogenic factors. It is characterized by the infiltration of inflammatory cells, the proliferation of fibroblasts, and the deposition of fibrous connective tissue in the lung interstitium (Todd et al., 2012; Rajasekaran et al., 2015; Chanda et al., 2019). The COVID-19 outbreak at the end of 2019 is one of the most devastating events in recent years. To date, more than 200 million people worldwide have recovered from COVID-19. However, although the virus has been eradicated, the infected patients still have varying degrees of pulmonary fibrosis complications, which can cause respiratory failure. In severe cases, these complications can lead to death (George et al., 2020). Several clinical, radiological, and histopathological research data indicated that secondary pulmonary fibrosis will threaten survival and cause exacerbation of functional lung damage in patients with severe COVID-19 (Lin et al., 2020; Schwensen et al., 2020; Tian et al., 2020; Zhou et al., 2020). At present, pirfenidone and nintedanib are the most promising drugs for the treatment of COVID-19-induced pulmonary fibrosis. Nintedanib is a transforming growth factor-β (TGF-β) inhibitors, as well as a chemosynthetic indole alkaloid. This is a breakthrough drug in the treatment of pulmonary fibrosis. Oral nintedanib can slow the progression of fibrosis and prolong life, but it has side effects, such as diarrhea and vomiting (Karimi-Shah and Chowdhury, 2015; Kim and Keating, 2015; Wollin et al., 2015). Therefore, with the further study of pulmonary fibrosis, it is extremely urgent to find more efficient and safer drugs to treat pulmonary fibrosis. The occurrence of pulmonary fibrosis is related to the overexpression of IL-1β, TNF-α, IL-6, IL-11, and other pro-fibrotic cytokines and the activation of some inflammatory signaling pathways (Karampitsakos et al., 2017; Li and Kan, 2017). Inhibiting the production of these mediators can significantly alleviate pulmonary fibrosis. At present, pulmonary fibrosis is treated using anti-inflammatory drugs, anti-fibrosis enzyme inhibitors, antioxidant mesenchymal stem cell therapy, and others.

**FIGURE 3:**
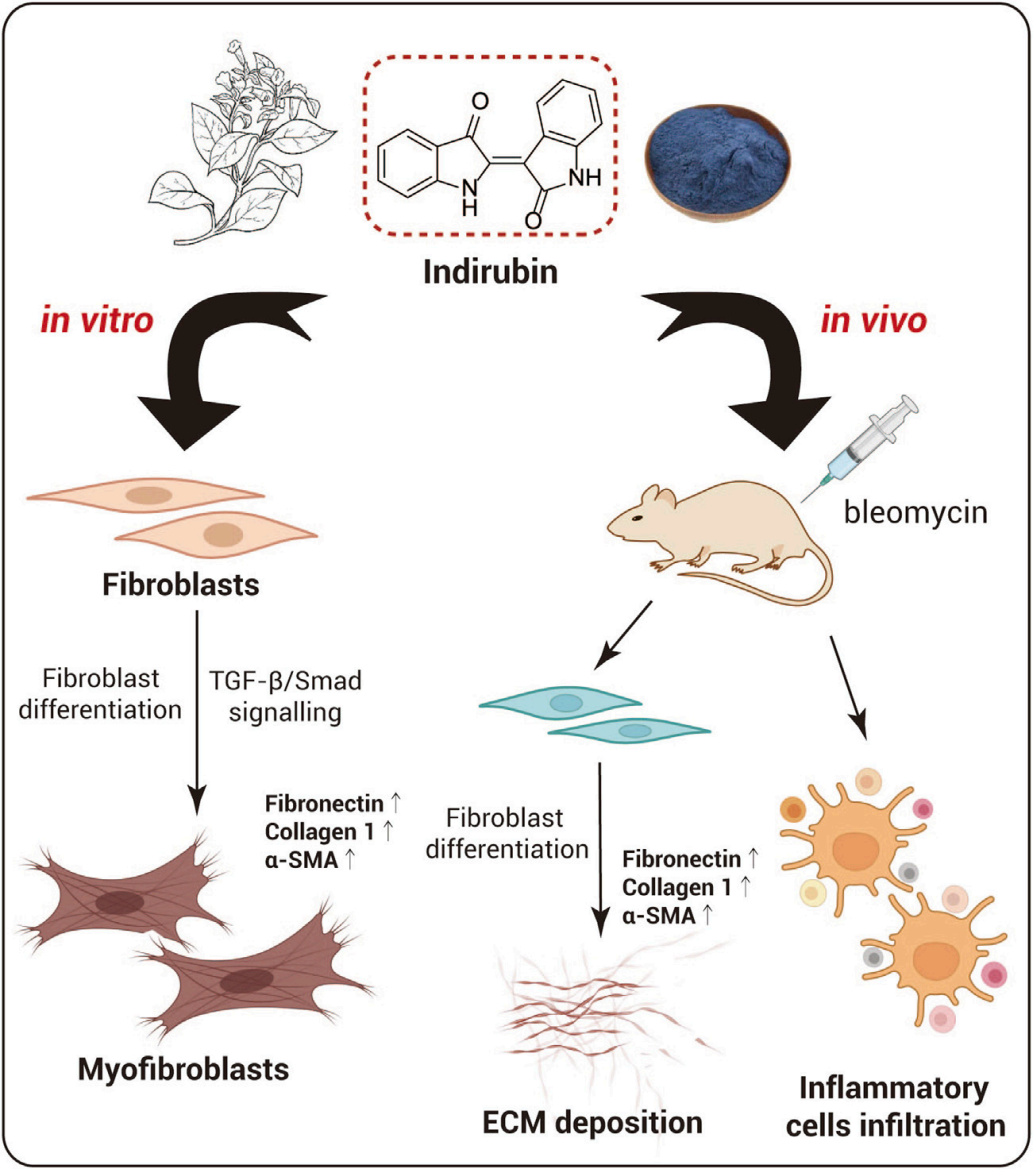
Indirubin ameliorates bleomycin-induced pulmonary fibrosis.

Indirubin, a bis-indole alkaloid, is extracted from indigo plants or mollusks of the *Muricidae* family. It has anti-tumor and anti-inflammatory effects (Gaboriaud-Kolar et al., 2015). Wang et al. investigated the effect of indirubin on bleomycin-induced pulmonary fibrosis in mice. Notably, Indirubin shows protective effect on lungs and can suppress the differentiation of fibroblasts to myofibroblasts in a dose-dependent manner, ultimately reducing BLM-induced lung damage and fibrosis. Indirubin significantly attenuated the expression of fibronectin, collagen I and α-SMA by inhibiting TGF-β/Smads signaling pathway. Results suggests that indirubin could be a good candidate drug for IPF treatment (Figure 3; Wang et al., 2020). Another indole alkaloid with potential is isorhynchophylline (isorhy), which was isolated from the traditional Chinese herb *Tripterygium wilfordii*. It reportedly shows anti-inflammatory activities in the nervous and cardiovascular systems (Zhou and Zhou, 2012). Qiu et al. found that isorhy could alleviate SD-induced pulmonary inflammation and fibrosis in mice by suppressing the release of IL-1b, TNF-α and IL-6 fibrogenic factors and notably reducing collagen deposition in lung tissues. Mice treated with isorhy showed alleviation in body weight loss induced by SD. In addition, isorhy treatment substantially assuaged inflammatory cell infiltration and fibroblast excessive proliferation (Qiu et al., 2020).

Zhao et al. conducted a study on the active ingredients in total alkaloids (TA) and their potential mechanism in the treatment of pulmonary fibrosis. The research suggested that through the analysis of cytokine, histopathological examination and gene expression, the treatment with 50 mg/kg TA could ameliorate pathological changes in the lung tissue, reduce the content of Krebs von den Lungen-6, TGF-β, collagen I and hydroxyproline in lung tissues, and increase the level of superoxide dismutase in the serum (Zhao et al., 2020). According to the above experimental data, the indexes of picrinine and scholaricine are superior to those of other alkaloids; thus, they are the most effective ingredients of TA in the treatment of PF. Overall, TA protected mice against BLM-induced fibrosis by enhancing the expression of TGF-β/matrix metalloproteinase-1 (MMP-1) pathway and diminishing the accumulation of collagen (Zhao et al., 2021). Kim et al. reported that I6CA, a natural indole derivative isolated from the marine brown algae *Sargassum thunbergia*, can protect V79-4 lung fibroblasts from oxidative stress. This group used H_2_O_2_ to induce oxidative damage and found that H_2_O_2_ can stimulate G2/M cell cycle arrest and DNA damage to reduce V79-4 cell survival. I6CA can reverse the cytotoxicity of H_2_O_2_ by reducing the accumulation of ROS. Additionally, I6CA could significantly promote Nrf2 expression and increase the activity of HO-1. A HO-1 inhibitor, zinc protoporphyrin IX, can suppress HO-1 activity and eradicate the ROS scavenging activity of I6CA, as well as prevent anti-apoptotic effects of I6CA (Kim and Choi, 2020). In an anti-fibrotic screening of indole alkaloid compound library, Li et al. found that matrine derivative compound 3f could attenuate idiopathic pulmonary fibrosis via suppression of fibroblast-to-myofibroblast transition and inhibition of the TGF-β/Smad signaling pathway. Furthermore, compound 3f exhibited approximately 266-fold higher anti-fibrotic activity against MRC-5 cell lines than matrine (Li et al., 2019b).

Ye et al. recently discovered evodiamine in *Evodia rutaecarpa*; this is an indole alkaloid with excellent anti-inflammatory effect. Evodiamine alleviated inflammation and pulmonary fibrosis induced by lipopolysaccharide (LPS) both *in vivo* and *in vitro* (Ye et al., 2021). After treating LPS-induced mice with 10 mg/kg evodiamine, the apelin level increased significantly, thereby suppressing the increase in the concentrations of IL-6 and CCL17. Therefore, the group speculated that apelin could play a crucial role in the molecular mechanism of evodiamine. In addition, evodiamine could inhibit cell apoptosis and stimulate apelin pathway to regulate the release of inflammatory factors and inhibit the development of inflammation, ultimately attenuating lung fibrosis in LPS-induced mice (Ye et al., 2021). Indole-3-carbinol (I3C), an indole alkaloidal compound extracted from cruciferous vegetables, reported that by activating the AhR-responsive genes in rat lungs, the pup’s lung injury induced by hyperoxia-hypoxia was alleviated, thereby improving alveolarization and decreasing fibrosis. In their experiments, I3C could activate TNF-α and NF-κB pathway to promote the expressions of VEGF, MCP1, MMP-8, and IL-6 and regulate inflammatory processes. According to results of histopathological examination, the degree of alveolitis and pulmonary fibrosis decreased in the I3C-treated group (Guzman-Navarro et al., 2021).

In a clinical study, nintedanib, a tyrosine kinase inhibitor, was used by Umemura et al. in an interventional trial to assess its efficacy and safety in COVID-19 treatment. The experiment was conducted on adult COVID-19 patients requiring mechanical ventilation (Umemura et al., 2021). The research showed that compared with the control group, the P/F ratio of nintedanib group was remarkably increased, the mechanical ventilation time was significantly shortened, and the volume of high-attenuation areas on CT images was notably decreased. These results demonstrated that nintedanib can minimize respiratory sequelae of COVID-19 and improve lung injury by regulating pulmonary fibrosis. Nintedanib can be a novel anti-fibrotic agent approved by the FDA to reduce fibrosis symptoms (Umemura et al., 2021). PXS-5120A is an indole-based fluoroallylamine inhibitor, which could inhibit LOXL2/3 (a secreted enzyme that catalyze the formation of cross-links in collagen and elastin) to treat lung and liver fibrosis. LOXL2 is overexpressed in patients with fibrosis; it usually regulates the TGF-β/Smad signaling pathway to activate lung fibroblasts. Therefore, LOXL2 could be a promising therapeutic target for lung fibrosis (Findlay et al., 2019).

### Myocardial Fibrosis

Myocardial fibrosis (MF) is a pathological process in which fibroblasts (CFs) in normal myocardial tissue proliferate and transform into myofibroblasts due to various pathological factors, resulting in excessive deposition of extracellular matrix, increased collagen concentration, and disordered collagen ratio (Kong et al., 2014; Rienks et al., 2014; Pinto et al., 2016). Myocardial fibrosis is closely related to many clinical cardiovascular diseases such as ischemic cardiomyopathy, diabetic cardiomyopathy, hypertensive heart disease, chronic heart failure and hypertrophic cardiomyopathy. As a common pathological result of various diseases, myocardial fibrosis eventually lead to increased myocardial stiffness, decreased ventricular diastolic function, severe ventricular arrhythmias, and even sudden death. According to WHO, cardiovascular disease is the leading cause of death in the world (van Nieuwenhoven and Turner, 2013; Leask, 2015; Kurose and Mangmool, 2016). The occurrence of myocardial fibrosis is closely related to regulatory cytokines (such as TGF-β, CTGF, and MMP), inflammatory factors (such as TNF-α, IL-1β, IL-6, and IL-10), oxidative stress and other factors that affect the occurrence and development of myocardial fibrosis by regulating the corresponding signaling pathways.

Yao et al. explored the anti-fibrosis effect of 3,3-diindolymethane (DIM) against adriamycin-induced cardiac fibrosis. DIM was obtained from a naturally food additive. Adriamycin possesses strong cardiotoxicity and can cause cardiac fibrosis, eventually leading to heart failure. The DIM notably showed anti-fibrotic effect on adriamycin-induced cardiac tissue; the effect involved the reduction of collagen I and α-SMA, the upregulation of breast cancer type 1 susceptibility protein (BRCA1), and the activation of transcription factor nuclear factor (erythroid-derived 2)-like 2 (Nrf2). Then, DIM alleviated oxidative stress in inflammatory tissues (Yao et al., 2013). Rutaecarpine is an indolopyridoquinazoline alkaloid with powerful cardiovascular effects; it can be isolated from *E. rutaecarpa*. Li et al. investigated the anti-fibrotic properties of rutaecarpine against right ventricular (RV) remodeling on rats (Li et al., 2016). The experiment data suggested that rutaecarpine could reduce the expression of several related factors (such as α-SMA, collagen-I, collagen-III, eIF3a, TGF-β1, and others) to attenuate the effect of hypoxic-induced RV remodeling in rats in a dose-dependent manner. Moreover, calcitonin gene-related peptide (CGRP) could attenuate the activity of TGF-β1. The results proved that the inhibitory effect of rutaecarpine on hypoxic-induced RV remodeling is stimulated by the release of CGRP, and this effect may also be associated with the eIF3a/p27 pathway (Li et al., 2016).

A study was designed and conducted by Wu et al. to evaluate the anti-myocardial fibrosis activities of evodiamine, an indole alkaloid isolated from the *E. rutaecarpa*. Evodiamine could suppress TGF-β1-induced activation of adult rat cardiac fibroblasts, which was evidenced by the decrease in the expressions of α-SMA, CTCF, and collagen I/III. In addition, evodiamine could suppress the migration ability of HUVECs (Wu et al., 2017b). Isorhynchophylline (isorhy) is an indole alkaloid obtained from *Uncaria rhynchophylla*. Zhang et al. found that isorhy prevented phenylephrine (PE)-induced myocardial hypertrophy and alleviated myocardial fibrosis in rats by inhibiting the expressions of TGF-β1, CTGF, and collagen I/III and other related fibrosis factors. These effects were associated with Nrf2 nuclear translocation and MAPK pathway (Zhang et al., 2020b).

**FIGURE 4:**
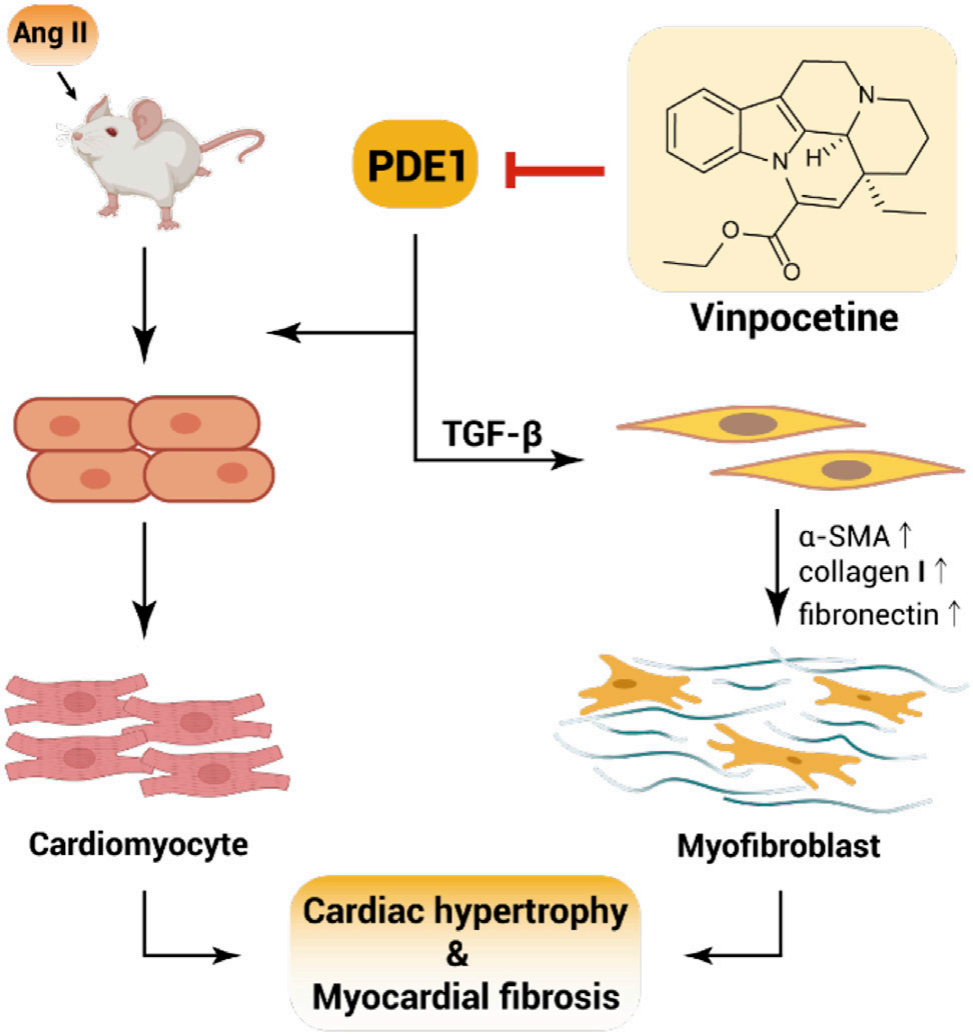
Vinpocetine attenuates Ang II-induced cardiac hypertrophy and myocardial fibrosis.

Another study performed by Wu et al. reported that vinpocetine is a derivative of vincamine alkaloid that is derived from periwinkle plant (*Vinca minor*). Vinpocetine improved cardiac hypertrophy and fibrosis induced by Ang II infusion (Wu et al., 2017a). The administration of vinpocetine at 5 mg/kg/day prevented heart enlargement by Ang II. Vinpocetine also inhibited the myocyte hypertrophic growth induced by Ang II *in vivo* and blocked the activation of fibroblasts and the expression of matrix gene stimulated by TGF-β *in vitro*. PDE1 was a potential molecular target that attenuates cardiac hypertrophy and fibrosis. Vinpocetine possesses critical pharmacological effects and can be used to treat cardiac remodeling and fibrosis (Figure 4, Wu et al., 2017a). Similarly, Liu et al. reported that meisoindigo (Me), a bisindole indirubin derivative, is widely used to treat chronic myelogenous leukemia. A study group assessed whether Me could improve myocardial cell damage and myocardial fibrosis in Streptozotocin (STZ)-induced type 1 diabetic rats. The outcomes of this study indicated that Me inhibited the STZ-induced production of pro-inflammatory mediators, including TNF-α and IL-2, by suppressing the activation of NF-κB and Wnt/β-catenin/GSK3β signaling pathways to alleviate cardiomyocyte hypertrophy and fibrosis (Liu et al., 2020).

Vincristine (VCR), a major vinca alkaloid, is commonly used in the treatments of various cancers, including lymphoma, leukemia, neuroblastoma, and so on (Said and Tsimberidou, 2014). Previous studies showed that VCR exerts cardioprotective effects on adult mouse myocytes or myocardial necrosis in rats. However, its efficacy on cardiac fibrosis treatment remains unclear (Panda et al., 2014). Therefore, Ge et al. conducted an experiment, in which ISO-injected adult male Sprague–Dawley (SD) rats were treated with VCR or vehicle. They found that VCR-treated rats showed further alleviation in the degree of myocardial fibrosis compared with the vehicle-treated rats, as demonstrated by the reduced heart/body weight ratio. The decreased colocalization among the nucleotide-binding domain, leucine-rich repeat, NLRP3, and ASC in VCR-treated rats could be observed. VCR could ameliorate cardiac fibrosis by down-regulating the expressions of caspase-1, IL-1β, and IL-18 and then inhibiting the activation of the NLRP3 inflammasome directly (Ge et al., 2021). 6-Bromoindirubin-3′-oxime (6BIO), an indirubin derivative, could attenuate cardiac fibrosis by increasing the anti-aging effects on aging heart. 6BIO promoted autophagy by decreasing the expression of p62 protein, increasing beclin-1 level and LC3II/I ratio, inhibiting the ROS production, and eventually alleviating oxidative stress. In addition, 6BIO may suppress the GSK3β and mTOR pathway to delay the aging process of an aging heart (Guo et al., 2020).

Carvedilol is a nonselective third-generation β-adrenoceptor that has been used to treat myocardial injury and fibrosis induced by acute myocardium infarction (AMI) and diabetic cardiomyopathy (DCM) (Zhu et al., 2013). For example, in *in vitro* experiments, Zhu et al. reported that carvedilol suppressed the activation of ROS-induced Smad3 to reduce Colla1, Col3a1, and α-SMA expressions and increase miR-29b expression in a dose-dependent manner. These data suggested that carvedilol protected rat against AMI-induced myocardial fibrosis through the abrogation of Smad3 and the upregulation of miR-29b (Zhu et al., 2013). Okumura et al. investigated the effects of carvedilol on biventricular fibrosis and its functions against pulmonary arterial hypertension (PAH) in a rat model. They reported that carvedilol decreased the concentration of collagen and the expression of TGF-β1 and CTGF. Moreover, the hemodynamics and exercise endurance improved. Carvedilol could ameliorate biventricular fibrosis by blocking the TGFβ1-CTGF pathway (Okumura et al., 2015). A study conducted by Zheng et al. found that carvedilol could elevate cardiac function and further improve myocardial fibrosis against DCM rats by upregulating the AKT/XIAP antiapoptotic pathway and inhibiting myocardial inflammation (Zheng et al., 2017). Melatonin is an indoleamine produced by the pineal gland. It possesses a wide range of pharmacological effects on various tissue and organs and prevents myocardial fibrosis (Song et al., 2020). Several studies reported the protective activities of melatonin against cardiac fibrosis. In a study where diabetic mice were used as research objects, Zhou et al. reported a novel pathway to induce the occurrence of diabetic cardiomyopathy. This disease was reportedly alleviated through the suppression of the activation of Syk/COX-1/SERCA axis by melatonin. Thus, it attenuated cardiac fibrosis and retained the vitality of cardiomyocytes (Zhou et al., 2018). In another study, Che et al. used diabetic mice and found that melatonin could significantly elevate cardiac dysfunction and exert antifibrotic effect by inhibiting the TGF-β1/Smad pathway and activating the NLRP3 inflammasome (Che et al., 2020). Similarly, Jiang et al. showed that the administration of melatonin at 20 mg/kg for 4 weeks could relieve PM_2.5_-induced cardiac dysfunction and fibrosis in mice. Further research showed that melatonin could suppress mitochondrial oxidative injury and regulate the deacetylation of SOD2 mediated by SIRT3 to exert anti-fibrotic activities (Jiang et al., 2021). These findings suggested that melatonin might be a promising agent for treating myocardial fibrosis.

### Renal Fibrosis

The main pathological characteristics of renal fibrosis are the activation and proliferation of fibroblasts, as well as the accumulation of extracellular matrix (ECM) deposited in the renal interstitium, which ultimately lead to the structural destruction and functional loss of renal tissue (Sun et al., 2016; Djudjaj and Boor, 2019). Renal fibrosis is a major pathological change and a common pathway of all chronic kidney diseases that progress to end-stage renal disease (Djudjaj and Boor, 2019). This condition in the kidney can be induced by multiple factors such as infection, injury, toxin and radiation, which cause the overproduction of free radicals and superoxide. These factors also stimulate the secretion of various cytokines, thus leading to the occurrence and development of renal fibrosis (Meng et al., 2014; Richter et al., 2015). TGF-β1, the key diver of renal fibrosis, induces the transformation of renal fibroblasts into myofibroblasts and promotes the fibrosis of renal diseases (Gu et al., 2020). TNF-α can induce the inflammation process; it is an essential pro-inflammatory mediator in the promotion of renal fibrosis. A large number of studies have confirmed that natural indole alkaloids and their synthetic derivatives can improve renal fibrosis and delay the progression of kidney disease by regulating a variety of cytokine-mediated signal transduction pathways (Meng et al., 2015).

**FIGURE 5:**
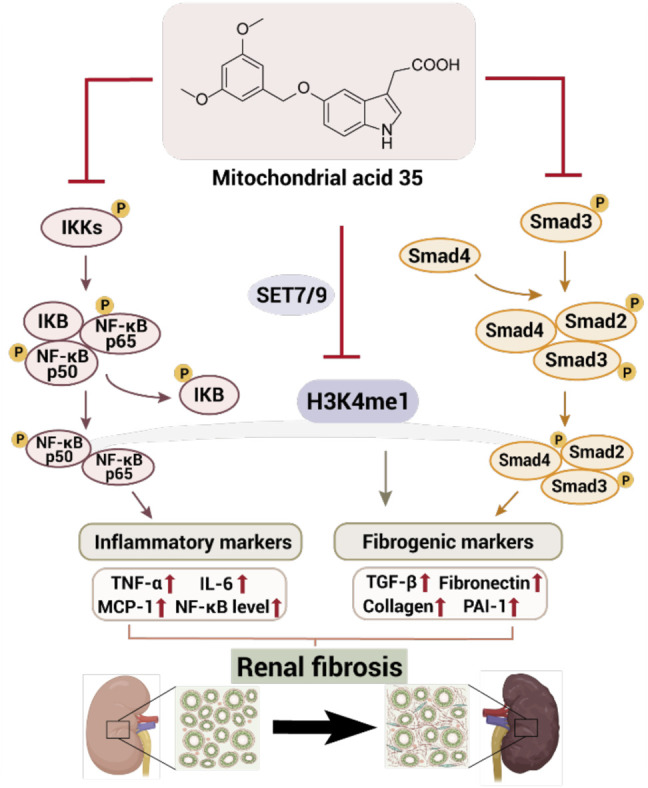
Mitochondrial acid 35 inhibits TNF-α and TNF-β1 pathway to ameliorate renal fibrosis.

According to Xia et al., 3,3-Diindolylmethane (DIM), obtained from cruciferous vegetables, inhibits the development of interstitial collagen fibrosis, fibronectin and collagen-1 expressions, and local fibroblast activation; it also decreases the phosphorylation of Smad2/3 and upregulates the expression of Smad7, eventually alleviating kidney injury and renal fibrosis. The antifibrotic effect of DIM on unilateral ureteral obstruction (UUO)-induced mice model was closely associated with the inhibition of TGF-β/Smad2/3 signaling (Xia et al., 2018). Shima et al. screened 11 indole derivatives with anti-TNF-α effect from an indole derivative library; mitochonic acid 35 (MA-35) exhibited not only anti-TNF-α effect but also anti-TGF-β1 effect by inhibiting the phosphorylation of Smad3 (Shima et al., 2017). MA-35 suppressed the phosphorylation of IκB kinase to exert anti-TNF-α activity in hepatic inflammation induced by LPS/GaIN in mice. In the unilateral ureter obstructed mouse model, MA-35 diminished renal inflammation and fibrosis by reducing the expression of inflammation factors and fibrotic genes, such as TNF-α, iNOS, MCP-1, and IL-6. Furthermore, MA-35 might affect the recovery of epigenetic modifications and inhibit TNF-α/IKK and TGF-β1/Smad3 signaling to attenuate renal fibrosis (Figure 5, Shima et al., 2017).

Yohimbine, an α2-adrenoceptor inhibitor from the dried bark extract of *Corynante Yohimbe*, showed renoprotective activities, according to Hayashi et al. (Hayashi et al., 2021). Results of experiments on a 5/6 nephrectomy-induced chronic kidney disease (CKD) rat model, showed that treatment with yohimbine significantly decreased urinary protein excretion and noradrenaline content of renal venous plasma compared with hydralazine-treated animals. Yohimbine treatment could suppress the mRNA expression levels of TGF-β, collagen Ⅲ, and fibronectin, thus inhibiting renal fibrosis. Yohimbine has potential as a therapeutic drug for CKD (Hayashi et al., 2021). Nintedanib, a small-molecule tyrosine kinase inhibitor, is an FDA-approved drug that is used to treat idiopathic pulmonary fibrosis. Recently, it was reported that nintedanib has a moderating effect on renal fibrosis. Liu et al. stated that in the UUO-induced mouse model, the administration of 50 mg/kg nintedanib greatly reduced the activation of renal interstitial fibroblasts and ameliorated renal fibrosis. Additionally, nintedanib suppressed PDGFRβ, FGFR1, FGFR2, and VEGFR2 phosphorylation and blocked the expressions of STAT3, NF-κB, and Smad3 to diminish macrophage infiltration, thereby ultimately exerting anti-renal fibrosis effects (Liu et al., 2017). In another study, Bigaeva et al. used precision-cut kidney slices (PCKS) as an experimental model to study the effect of nintedanib on renal fibrosis. The study showed that nintedanib could inhibit the phosphorylation of PDGFR and VEGFR, suppress cell proliferation, and decrease the content of collagen-I and the expression of fibrosis-related genes, thereby protecting against the occurrence of fibrosis in both murine and human PCKS. However, it could not reverse fibrosis (Bigaeva et al., 2020).

Zhang et al. reported a novel target for treating renal fibrosis, namely, histone deacetylase 8 (HDAC8), which is expressed in renal tubular epithelial cells. In UUO-induced renal fibrosis mice model, the HDAC8 inhibitor, PCI34051, inhibited Smad3, STAT3, β-catenin and Snail expressions and activated BMP7 and Klotho renal protective protein expressions. In addition, fibrotic markers, including α-SMA, collagen 1, and fibronectin, could be inhibited, thereby alleviating the occurrence of renal fibrosis (Zhang et al., 2020c). SB-216763, as a GSK-3β inhibitor, could reverse the hypertrophy and dysfunction of the heart and kidneys in aldosterone (Aldo)-induced rats by suppressing the expression of inflammatory factors including TNF-a, IL-1β, and MCP-1. SB-216763 triggered the activation of autophagy by upregulating the LC3-II protein levels and promoting p62 protein degradation in cardiac and renal tissues, which played an important role in ameliorating perivascular fibrosis, and renal injury (Zhang et al., 2018).

A synthetic indole-contained compound, LG4, has been improved to possess anti-inflammatory activities both *in vitro* and *in vivo* (Liu et al., 2019b). Qian et al. found that LG4 has renal protective effects on diabetic kidney disease (DKD) in T1MD mice. Moreover, it could significantly ameliorate glomerulosclerosis and fibrosis induced by hyperglycemia in TIDM mice without affecting the body weight and blood glucose levels. These effects were manifested in the decreased expressions of COL-4 and TGF-β. Additionally, *in vivo* and *in vitro* data showed that LG4 could improve anti-inflammatory effects by suppressing the phosphorylation of JNK and ERK and by activating NF-κB signaling. However, the renal protective activity of DKD in type 2 diabetic mice needs further study (Qian et al., 2021). Tropisetron, a synthetic indole drug, has been used to inhibit chemotherapy-induced vomiting in clinic. Previous research showed that tropisetron has multiple pharmacological properties including anti-inflammatory, anti-diabetic, and anti-fibrotic (Barzegar-Fallah et al., 2015). In this study, Pourheydar et al. investigated the anti-renal fibrosis effects of tropisetron against diabetic nephropathy rat model. The results showed that tropisetron could ameliorate kidney function and attenuate renal fibrosis by inhibiting the expressions of TGF-β1 and p53 proteins, as well increasing the level of extracellular matrix metalloproteinases, including MMP-9 and MMP-2 (Pourheydar et al., 2021).

Melatonin is a pineal hormone with strong anti-oxidant and anti-inflammatory effects. Recently, several studies showed that melatonin has a certain protective effect on kidney injury and anti-renal fibrosis, especially for the fibrosis of diabetic mice, which has been widely studied. In animal models of renal fibrosis caused by hyperglycemia, Li et al. reported that melatonin exerted anti-renal fibrosis activities, as shown by the restoration of mitochondrial function and the activation of the AMPK/PGC1α pathway (Li et al., 2019a). For aristolochic acid (AA)-induced nephropathy (AAN) mice model, the contents of blood urea nitrogen and creatinine decreased, and renal tubules dilated, but treatment with melatonin could reverse these indexes. Melatonin alleviated tubulointerstitial fibrosis by inhibiting the TGF-β/Smad pathway (Kim et al., 2019). Additionally, according to Li et al., melatonin was used to ameliorate TGF-β1-induced renal fibroblast to myofibroblast transdifferentiation (FMT) and UUO-induced renal fibrosis by decreasing the levels of α-SMA, collagen-Ⅰ, fibronectin, and miR-21-5p and the phosphorylation of STAT3 and by increasing the expressions of Spry1 and PTEN (Li et al., 2020a). Toon et al. found that melatonin is a promising drug for the treatment of a chronic kidney disease (CKD) patient with renal fibrosis; melatonin could recover mitochondrial function by upregulating the miR-4516 expression to decrease ROS formation (Yoon et al., 2020). In response to acute kidney injury induced by sepsis, melatonin could reduce the oxidative stress response and ROS accumulation of ROS *in vitro* and attenuate the inflammatory response by downregulating the mRNA expressions of IL-1α, IL-1β, Mcp-1 and TGF-β1, thereby proving the therapeutic effect of melatonin on acute kidney injury (Chen et al., 2021).

### Liver Fibrosis

Liver fibrosis (LF) is a self-injury repair reaction caused by chronic liver injury, including hepatitis virus infections, metabolic diseases, non-alcoholic steatohepatitis and immune liver injury. If liver fibrosis is not effectively controlled, then it will eventually develop into cirrhosis or lead to life-threatening liver cancer (Parola and Pinzani, 2019; Zhangdi et al., 2019). The formation of hepatic fibrosis includes the activation and transformation of hepatic stellate cells, collagen deposition, and remodeling of extracellular matrix proteins (Campana and Iredale, 2017). Hepatic fibrosis is reversible. Several indole alkaloid compounds regulate the TGF-β/Smad, NF-κB, MAPK, and other signaling pathways to inhibit oxidative stress and inflammatory response and then reduce liver injury to alleviate hepatic fibrosis. Hepatic stellate cells (HSCs) are the main cell sources of matrix components. They play an essential role in the occurrence and development of liver fibrosis (Novo et al., 2014; Higashi et al., 2017). Induction of HSC apoptosis is a potential way to reverse liver fibrosis (Elsharkawy et al., 2005). According to Li et al., indole-3-carbinol (I3C), a natural compound obtained from *Brassica* vegetables, could induce HSC apoptosis to attenuate hepatic fibrosis by increasing deubiquitinase cylindromatosis (CYLD) level and decreasing K63-ubiquitination of RIP1. These effects were closely related to the upregulation of Bax/Bcl-2 ratio and caspase-8, as well as the inhibition of the NF-κB pathway (Li et al., 2017). Zahran et al. demonstrated that phthalimide-indole analogs, including compound 8, effectively improved the anti-liver fibrotic activity by downregulating the Bcl-2 protein and upregulating caspase-3 to promote apoptosis. Compound 8 also possessed strong anticancer effect on HepG2, MCF-7, A549 and other cell lines. According to histopathological studies, compound 8 treatment could restore fibrotic liver tissue to normal (Zahran et al., 2018).

**FIGURE 6:**
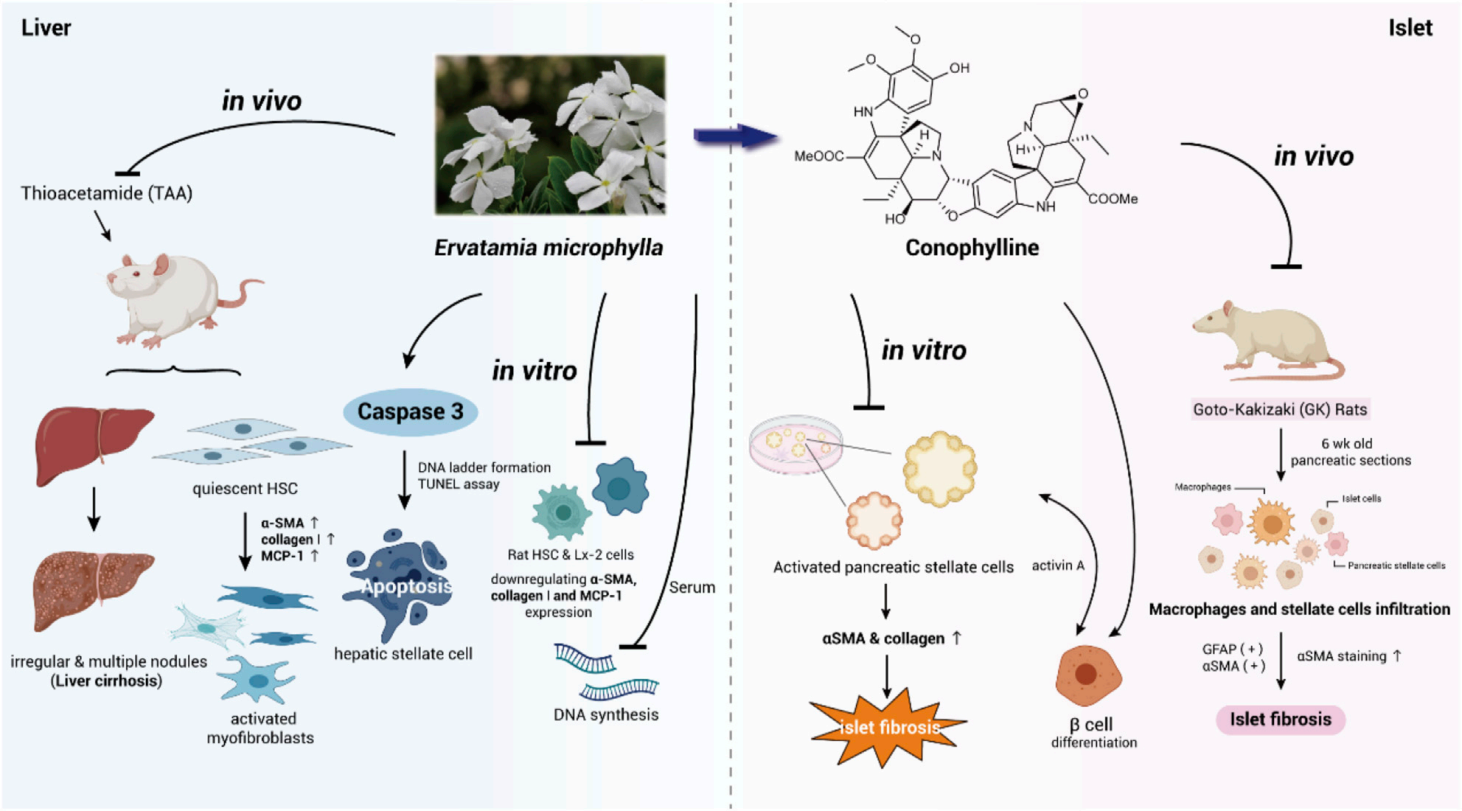
Conophylline inhibits activation of stellate cells to attenuate liver fibrosis and islet fibrosis.

Conophylline (CnP), an indole alkaloid extracted from tropical plant *Ervatamia microphylla,* reportedly attenuates liver fibrosis. In rat HSCs and Lx-2 cells, CnP decreased the α-SMA and collagen-1 expressions and DNA synthesis, as well as upregulated the caspase-3 to induce apoptosis. In thioacetamide-induced rats, the liver surface of rats was not smooth, and many nodules were present. CnP treatment reversed these phenomena, and the concentration of collagen decreased. CnP could be a promising compound for the treatment of hepatic fibrosis (Figure 6; Kubo et al., 2014). In Yang et al.’s study, evodiamine ameliorated liver fibrosis by inhibiting the TGF-β1/Smad pathway. Evodiamine decreased the contents of IL-6, TNF-α, and collagen-Ⅰ/Ⅲ. The level of TGF-β1, phosphorylation of Smad 2/3, and α-SMA were blocked by evodiamine in CCl_4_-induced liver fibrosis rat model. Meanwhile, evodiamine suppressed cell proliferation, hydroxyproline production, and collagen metabolism in HSCs in a dose-dependent manner (Yang et al., 2018). These data proved that evodiamine might be a potential therapeutic drug for treating fibrotic liver diseases (Li et al., 2020b).

Inflammatory cytokines play an important role in initiating the activation and regulation of liver fibrosis. Elnfarawy et al. tested the anti-fibrotic activity of a synthetic vinca alkaloid vincamine derivative, namely, vinpocetine against thioacetamide-induced liver fibrosis rat model. By downregulating the hydroxyproline and α-SMA level, the liver oxidative stress and histopathological damage were significantly improved. This group discovered that vinpocetine could inhibit angiogenesis and proliferation by reducing VEGF/KI-67 expression (Elnfarawy et al., 2021). Cyclic adenosine monophosphate (cAMP) can inhibit the proliferation of fibroblasts and the synthesis of ECM protein. The activity of cAMP is regulated by phosphodiestrases (PDEs) to some extent (Insel et al., 2012). To explore the mechanism of a PDE inhibitor in liver fibrosis by using vinpocetine as the PDE-1 inhibitor, Essam et al. studied its therapeutic effect in diethylnitrosamine (DEN)-induced liver fibrosis. Their results showed that TNF-α, TLR4, and TIMP-1 expressions decreased and CREB protein expression increased remarkably. Furthermore, the concentrations of hydroxyproline, TGF-β1 and NF-κB were significantly inhibited (Essam et al., 2019). Additionally, Alhusseiny et al. reported that vinpocetine can also be used as a novel adjuvant of praziquantel to attenuate liver fibrosis of schistosoma (Alhusseiny et al., 2018).

VD60 is a novel indol-contained skeleton CB_1_ inhibitor. Wei et al. investigated the anti-hepatic fibrosis effects of VD60 *in vitro* and *in vivo* (Wei et al., 2014). In the human HSC line LX-2 and rat HSCs, VD60 inhibited the expression of α_2_(I) procollagen mRNA, the production of ROS, and the phosphorylation of Akt, ERK, and Smad3, thereby exerting a remarkable anti-proliferation activity. In CCl_4_-induced liver fibrosis mouse model, the VD60-treated group showed that a-SMA expression, TGF-β, and fibronectin mRNA levels, the fibrotic area, and hepatic hydroxyproline (HYP) deposition all decreased compared with the vehicle group, suggesting that VD60 could effectively ameliorate liver fibrosis. They measured VD60 concentration in the plasma and brain, and the results showed that the content of VD60 in blood was much higher than that in brain. This finding indicated that VD60 could not penetrate the blood-brain barrier and played an anti-fibrosis role as a peripheral CB1 antagonist (Wei et al., 2014). Wu et al. discovered that carvedilol retarded the cell cycle at the G0/G1 phase and decreased the α-SMA expression and collagen I/III deposition to inhibit the proliferation of HSC induced by Ang II, thereby ameliorating hepatic fibrosis. Additionally, carvedilol promoted HSC apoptosis by down-regulating Bcl-2 protein expression and attenuated liver fibrosis (Wu et al., 2019). Indole-3-carboxaldehyde (3-IAld), an indole metabolite, was produced from commensal *Lactobacillus reuteri*. 3-IAld promoted the recovery of intestinal barrier function and played an important role in the treatment of liver and gastrointestinal diseases. In the DCC-induced PSC murine model, Onofrio et al. found that 3-IAld could reduce the formation of liver fibrosis by inhibiting the expressions of TGF-β1 and IL-9. It maintained intestinal mucosa homeostasis by regulating intestinal flora and activating the microbiota aryl hydrocarbon receptor (AhR)-IL-22 axis, thus preventing and ameliorating liver inflammation and fibrosis (D’Onofrio et al., 2021). As an indoleamine compound, melatonin affects the activity of hepatic stellate cells and has an inhibitory effect on some pro-inflammatory factors, such as TNF-α, IL-1β, IL-6, and TGF-β1. In some experimental animal models of hepatic fibrosis, melatonin regulates oxidative stress, inflammatory response, and apoptosis to prevent and treat liver injury and liver fibrosis caused by various factors (Kang et al., 2016; Gonzalez-Fernandez et al., 2017; Mortezaee et al., 2017; Lebda et al., 2018).

### Islet Fibrosis

Islet fibrosis occurs in patients with type 2 diabetes mellitus (T2DM). It may interfere with the metabolism of pancreatic β-cells, thereby affecting insulin secretion, destroying the normal structure of islet, and promoting the occurrence of T2DM (Katsuda et al., 2014). The pathogenesis of islet fibrosis may be related to the activation of pancreatic stellate cells (PSCs). Under the stimulation of chronic pancreatitis and pancreatic cancer, PSCs are activated to express α-smooth muscle actin (α-SMA), secrete collagen, and synthesize amounts of extracellular matrix proteins, resulting in islet fibrosis (Lee et al., 2017; Xue et al., 2018). Goto-Kakizaki (GK) rats are among the most characteristic animal models of spontaneous type 2 diabetes mellitus, which is characterized by a decrease in the number of pancreatic β-cells and fibrosis formation. The metabolism and diabetes indicators of GK rats are very similar to human T2DM. Thus, they can be used for the study of islet fibrosis (Homo-Delarche et al., 2006).

**FIGURE 7:**
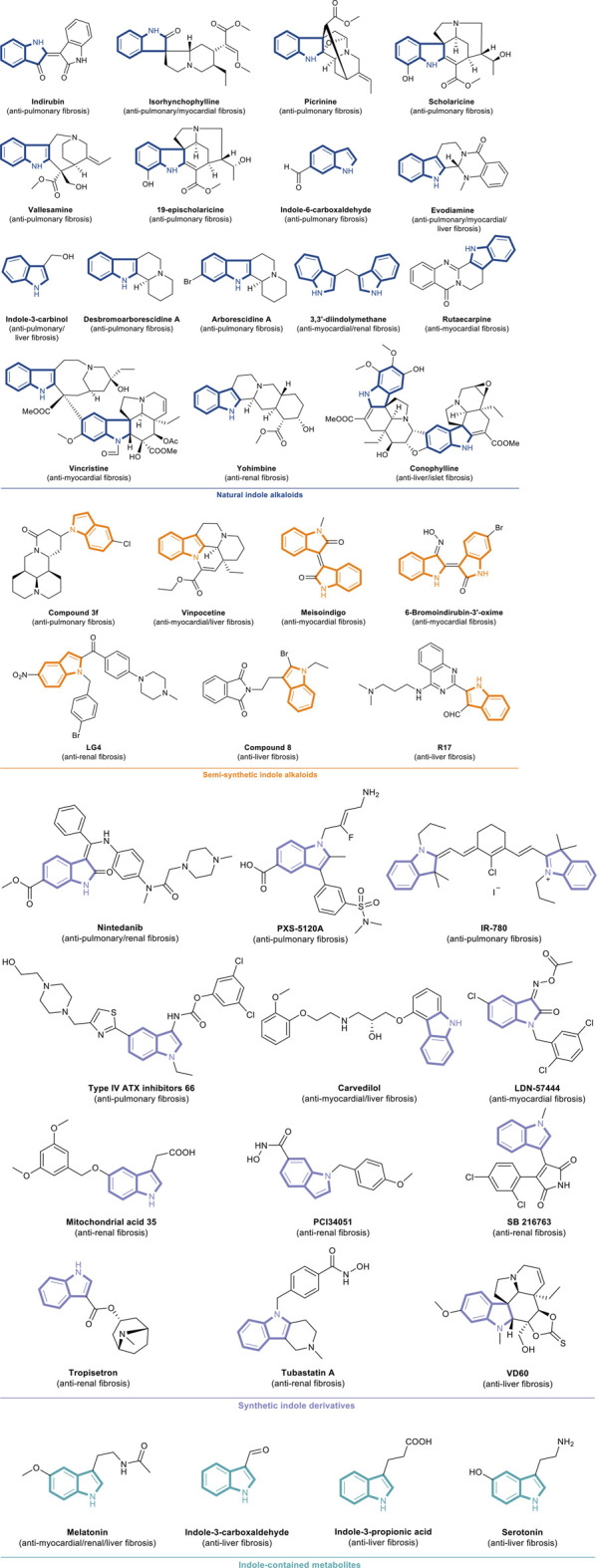
(*Continued*) The chemical structures of anti-fibrotic indole alkaloids and indole derivatives.

In a study by Saito et al., conophylline (CnP), a natural indole alkaloid, obtained from the leaves of *E. microphylla*, reportedly exerts anti-islet fibrosis activity against Goto-Kakizaki rat model (Saito et al., 2012). CnP could suppress the activation of stellate cells and decrease the collagen-Ⅰ production. In 6-week-old GK rat pancreatic sections, the CD68-positive macrophages and GFAP- and α-SMA-positive stellate cells infiltrated into islets, and subsequently, macrophages and α-SMA stained stellate cells increased. This finding suggested that stellate cells were involved in islet fibrosis in GK rats. In *in vivo* experiments, after treating GK rats with 0.9 μg/g dose of CnP for 4 weeks, the invasion of PSCs and macrophages decreased significantly, and the concentrations of insulin elevated greatly. All the abovementioned data proved that CnP is a promising compound for ameliorating islet fibrosis both *in vitro* and *in vivo* (Figure 6; Saito et al., 2012). In another study, conophylline inhibited the activity and proliferation of cancer-associated fibroblasts (CAF) and reduced the desmoplasia of cancer tissue. It suppressed tumor proliferation when combined with anti-tumor drug, gemcitabine (Ishii et al., 2019). A brief illustration of all indole alkaloids is presented in Figure 7 and Table 1.

**TABLE 1:** Therapeutic activities of indole alkaloids and indole derivatives on organ fibrosis.

Disease	Compound	Source	Study model	Stimulus	Dosage	Activity/targets/pathway	References
Pulmonary fibrosis	Indirubin	*Muricidae*	C57BL/6 mice	Bleomycin	12.5 and 25 mg/kg	TGF-β/Smad ↓	Wang et al. (2020)
	Isorhynchophylline (isorhy)	*Uncaria rhynchophylla*	Mice	SiO_2_	20 mg/kg	TGF-β1, TNF-α, IL-1β, IL-6 ↓ collagen deposition ↓	Qiu et al. (2020)
	Picrinine	*Alstonia scholaris*	Mice	Bleomycin	5 mg/kg	TGF-β/MMP-1 ↓	Zhao et al. (2020); Zhao et al. (2021)
	Scholaricine				3 mg/kg	TGF-β/MMP-1 ↓	
	Vallesamine				3 mg/kg	IL-11, MMP-12, TGF-β ↓	
	19-epischolaricine				1 mg/kg	IL-11, MMP-12, TGF-β ↓	
	Indole-6-carboxaldehyde (I6CA)	*Sargassum thunbergii*	*in vitro*	H_2_O_2_	300 μM	Nrf2/HO-1 ↑	Kim and Choi (2020)
	Compound 3f	Matrine derivative	MRC-5 cell lines	—	3.3 ± 0.3 μM (IC50)	TGF-β/Smad pathway ↓	Li et al. (2019b)
	Evodiamine	*Evodia rutaecarpa*	BALB/c mice	Lipopolysaccharide	10 mg/kg	Apelin ↑	Ye et al. (2021)
	Indole-3-carbinol (I3C)	*Brassica* vegetables	Rat pups	Hyperoxia–hypoxia	100 mg/kg	MMP-8, IL-6, NF-κB ↓	Guzman-Navarro et al. (2021)
	Nintedanib	Synthetic compound	Human	COVID-19	150 mg (twice daily)	α-SMA, S100A4, COL1, FN ↓	Umemura et al. (2021)
	PXS-5120A	Synthetic compound	C57BL/6 mice	Bleomycin/CCl_4_	20 mg/kg	LOXL2/3 ↓	Findlay et al. (2019)
	Desbromoarborescidine A	*Dracontomelum mangiferum*	*in vitro*	—	82.5 μM (IC_50_)	showed cytotoxic activity	Santos et al. (2009)
						towards human lung	
						fibroblast cells	
	Arborescidine A	*Pseudodistoma arborescens*	*in vitro*	—	71.6 μM (IC_50_)	showed cytotoxic activity	Santos et al. (2009)
						towards human lung	
						fibroblast cells	
	Type IV ATX inhibitors 66	Synthetic compound	C57Bl/6J mice	Bleomycin	20 or 60 mg/kg	exerted a high inhibition of the *ex vivo* ATX activity	Lei et al. (2020)
					0.43 nM (IC_50_)		
	IR-780	Synthetic compound	*in vitro*	Radiation	0.4 mg/kg	collagen I, α-SMA ↓	Luo et al. (2021)
			C57BL/6 mice				
Myocardial fibrosis	3,3′-diindolymethane (DIM)	Cruciferous plants	*in vivo*	Adriamycin	2.5 mg/kg	BRCA1 ↑ Nrf2 ↑	Yao et al. (2013)
						Collagen I ↓ α-SMA ↓	
	Rutaecarpine	*Evodia rutaecarpa*	Rats	Hypoxia	20 or 40 mg/kg	α-SMA, TGF-β1 ↓	Li et al. (2016)
	Evodiamine	*Evodia rutaecarpa*	Neonatal rats	TGF-β1	0.1, 1, 5, 10 μM	TGF-β1/Smad ↓	Wu et al. (2017b)
						α-SMA, collagen-I/III, CTGF ↓	
	Isorhynchophylline (isorhy)	*Uncaria rhynchophylla*	C57BL/6 mice	Phenylephrine (PE)	0, 5, 10, 25, 50 μM	TGF-β1, CTGF, collagen I/III ↓	Zhang et al. (2020b)
	Vinpocetine	Vincamine derivative	C57/BL6 male mice	Ang II	5 mg/kg	α-SMA, collagen-1, ECM ↓	Wu et al. (2017a)
	Meisoindigo (Me)	Indirubin derivative	Type 1 Diabetic Rats	Streptozotocin (STZ)	20 mg/kg	NF-κB ↓	Liu et al. (2020)
						Wnt/β catenin/GSK3β ↓	
	Vincristine (VCR)	*Catharanthus roseus*	Sprague-Dawley (SD) rats	Isoprotereno (ISO)	25 and 50 μg/kg	NLRP3 ↓ caspase-1, IL-1β, IL-18 ↓	Ge et al. (2021)
	6-Bromoindirubin-3′-oxime (6BIO)	Indirubin derivative	C57BL/6J mice	—	10 mg/kg	p62, beclin-1, LC3II/I, ROS ↓	Guo et al. (2020)
						GSK3β/mTOR pathway ↓	
	Carvedilol	Synthetic compound	Rats	ligating the left anterior descending	1, 5 and 10 mg/kg	Colla1, Col3a1, α-SMA ↓ miR-29b ↑	Zhu et al. (2013)
				coronary artery			
			Rats	Monocrotaline	15 mg/kg	TGFβ1-CTGF signaling ↓	Okumura et al. (2015)
			Rats	Streptozotocin (STZ)	10 mg/kg	AKT/XIAP ↑ caspase-3 ↓	Zheng et al. (2017)
	Melatonin	Pineal gland	Cardiac-specific Syk knockout mice	Streptozotocin (STZ)	20 mg/kg	Syk/COX-1/SERCA ↓	Zhou et al. (2018)
			Mice	Streptozotocin (STZ)	10 mg/kg	TGF-β1/Smad ↓ α-SMA ↓	Che et al. (2020)
						NLRP3 ↓	
			ApoE^−/−^ mice	PM_2.5_	20 mg/kg	Collagen I/III ↓ α-SMA ↓ SOD2 ↑	Jiang et al. (2021)
	LDN-57444	Synthetic compound	Spontaneously hypertensive rats (SHRs)	—	20 μg/kg	TGF-β/Smad 2/3 ↓	Han et al. (2020)
Renal fibrosis	3,3′-Diindolylmethane (DIM)	Cruciferous plants	*in vivo*/*in vitro*	Unilateral ureteral obstruction (UUO)	100 mg/kg	TGF-β/Smad2/3 ↓ Collagen-1 ↓	Xia et al. (2018)
	Mitochondrial acid 35 (MA-35)	Synthetic compound	LX-2 cells C57BL/6 mice	LPS/D-GaIN	80 mg/kg	TGF-β1, TNF-α, iNOS ↓ MCP-1, IL-6 ↓	Shima et al. (2017)
	Yohimbine	*Corynante Yohimbe*	Rats	5/6 nephrectomy	0.3 or 3.0 mg/L	TGF-β1 mRNA, collagen I ↓	Hayashi et al. (2021)
	Nintedanib	Synthetic compound	Male C57/Black mice	Unilateral ureteral obstruction (UUO)	50 mg/kg	STAT3, NF-κB, Smad3 ↓ PDGFRβ, VEGFR2 ↓	Liu et al. (2017)
			Precision-cut kidney slices (PCKS)	—	0.1–10 μM	PDGFR, VEGFR ↓ Collagen-1 ↓	Bigaeva et al. (2020)
	PCI34051	Synthetic compound	Murine	Unilateral ureteral obstruction (UUO)	20 mg/kg	α-SMA, collagen ↓ TGF-β1/Smad3 ↓ STAT3 β-catenin ↓	Zhang et al. (2020c)
	SB 216763	Synthetic compound	Rats	Aldosterone (Aldo)	1.5 mg/kg	TNF-α, IL-1β, MCP-1 ↓ LC3-II ↑ p62 ↓	Zhang et al. (2018)
	LG4	Indole-2-Carboxamide derivative	C57BL/6 mice	Streptozotocin (STZ)	5 and 10 mg/kg	TNF-α, IL-6, ↓ MAPK/NF-κB ↓	Qian et al. (2021)
	Tropisetron	Synthetic compound	Male Wistar rats	Streptozotocin (STZ)	3 mg/kg	TGF-β1, p53 ↓ MMP-9, MMP-9 ↑	Pourheydar et al. (2021)
	Melatonin	Pineal gland	Mice	Streptozotocin (STZ)	20 mg/kg	AMPK/PGC1α ↑	Li et al. (2019a)
			C57BL/6N mice	Aristolochic acid (AA)	20 mg/kg	TGF-β1/Smad ↓	Kim et al. (2019)
			NRK-49F cells C57BL/6 mice	TGF-β1/Unilateral ureteral obstruction (UUO)	20 and 50 mg/kg	STAT3, miR-21-5p ↓ PTEN, Spry1 ↑	Li et al. (2020a)
			TH1 cell BALB/c mice	P-cresol/0.75% adenine	1 μM for 24 h	miR-4516 ↑ ROS ↓	Yoon et al. (2020)
			C57BL/6 mice	Cecal ligation puncture (CLP)	25 μg/ml	IL-1α, IL-1β, Mcp-1 and TGF-β1, ROS ↓	Chen et al. (2021)
	Tubastatin A	Synthetic compound	*in vivo*/*in vitro*	Angiotensin II (ANG)	10 mg/kg	Smad2/3, TGF-β, CTGF, TNF-α ↓	Choi et al. (2015)
Liver fibrosis	Indole-3-carbinol (I3C)	*Brassica* vegetables	HSC-T6 cell	—	25, 50, and 100 μM	Bax/Bcl-2 ↑ CYLD ↑ RIP1 K63 de-ubiquitination ↑	Li et al. (2017)
	Compound 8	Phthalimide– analog	*in vitro*/*in vivo*	CCl_4_	10 mg/kg	Bcl-2 ↓ Caspase-3 ↑ Improve the fibrotic liver tissues to normality	Zahran et al. (2018)
	R17	Bouchardatine derivative	C57BL/6J mice	high fat (HF)	20 mg/kg	TGF-β, collagen I, MCP1 Smad3 ↓	Rao et al. (2019)
	Conophylline (CnP)	*Ervatamia microphylla*	BALB/c mice Lx-2 cells	Thioacetamide (TAA)	4.09 mg/ml	α-SMA, collagen-1 ↓ caspase-3 ↑	Kubo et al. (2014)
	Evodiamine	*Evodia rutaecarpa*	Male Wistar rats Hepatic stellate cells	CCl_4_	15 and 25 mg/kg	TGF-β1/Smad ↓ IL-6, TNF-α and collagen-Ⅰ/Ⅲ ↓	Yang et al. (2018)
	Vinpocetine	Vincamine derivative	Sprague-Dawley rats	Thioacetamide (TAA)	10–20 mg/kg	Hydroxyproline, α-SMA ↓ VEGF/KI-67 ↓	Elnfarawy et al. (2021)
			Male Wistar rats	Diethylnitrosamine (DEN)	10 mg/kg	TNF-α, TLR4, TIMP-1 TGF-β1 and NF-κB ↓ CREB ↑	Essam et al. (2019)
	VD60	Synthetic compound	*in vitro*/*in vivo*	CCl_4_	10, 15, and 25 mg/kg	α-SMA, TGF-β1 ↓ ROS, Akt, ERK, Smad3 ↓	Wei et al. (2014)
	Carvedilol	Synthetic compound	*in vitro*/*in vivo*	CCl_4_	10 mg/kg	α-SMA, collagen I/III, ACE1 ↓	Wu et al. (2019)
	Indole-3-carboxaldehyde (3-IAld)	*Lactobacillus reuteri*	C57BL/6 mice	3,5-diethoxycarbonyl-1,4-dihydrocollidine (DDC)	18 mg/kg	α-SMA, TGF-β, IL-9 ↓ AhR-IL-22 axis ↑	D'Onofrio et al. (2021)
	Indole-3-propionic acid (IPA)	*Clostridium Sporogenes*	LX-2 cells	TGF-β	100 μM	COL1A2, α-SMA ↓	Sehgal et al. (2021)
	Melatonin	Pineal gland	Rats	CCl_4_	2.5, 5, and 10 mg/kg	α-SMA, TGF-β ↓	Kang et al. (2016)
			Mice	CCl_4_	5 or 10 mg/kg	SphK1/S1P pathway ↓	Gonzalez-Fernandez et al. (2017)
			Rats	Thioacetamide (TAA)	5 mg/kg	thioredoxin-1 mRNA transcripts ↑	Lebda et al. (2018)
			*in vivo*/*in vitro*	CCl_4_	5 or 10 mg/kg	α-SMA, collagen-1 ↓ PPAR-α ↑	Mortezaee et al. (2017)
	Serotonin	Metabolite	C57BL/6 mice	Concanavalin A	—	TGF-β1/Smads ↓ IL-6, IFN-γ, TNF-α, TGF-β1 ↓	Pang et al. (2021)
Islet fibrosis	Conophylline (CnP)	*Tabernaemontana divaricata*	Male Goto-Kakizaki (GK) rats	—	0.9 μg/g	Collagen, α-SMA, MCP-1 ↓ PSCs ↓	Saito et al. (2012)

## Conclusion and Outlooks

The current review summarizes the recent advancements in the use of indole alkaloids and indole derivatives to treat organ fibrosis. Multiple organ damage can trigger complex cellular and molecular cascades that eventually lead to fibrosis. Some natural indole alkaloids show perfect anti-inflammatory, antiviral, and antibacterial effects. Thus, their medicinal value is beyond doubt and deserves more attention. In this review, through the analysis of the related targets and pathways of pulmonary, myocardial, renal, liver, and islet fibrosis, small molecules containing indole skeletons regulated TGF-β1/Smad, NF-κB, Wnt/β-catenin, Nrf2/HO-1, and other pathways to inhibit different types of fibrosis. Current reports showed that although many research groups have demonstrated the potential of indole alkaloids in the treatment of organ fibrosis through *in vivo* and *in vitro* trials, only a few clinical trials have been conducted. Therefore, further preclinical and clinical trials are needed to evaluate the efficacy of these natural and synthetic indole alkaloids and to determine whether they can be successfully used in the clinical treatment of human organ fibrosis. In the future, we hope that more indole alkaloids can be found and synthesized. Clinical studies can be carried out on these indole alkaloids. Their structures can be modified and transformed appropriately to improve the efficacy. Currently, one of the major obstacles of translating novel antifibrotic drugs to the clinic is due to the intricacies of drug delivery. We may be able to load such small molecules on nanoparticles to improve drug stability and targeting, and reduce unwanted cellular uptake. Treatment using nanoparticle systems is a promising tool for the therapy of chronic inflammatory diseases, which provides new perspectives and ideas for the treatment of fibrosis. These future prospects can lead to certain strategies for the development of new targeted fibrosis drugs.

## Data Availability

The original contributions presented in the study are included in the article/Supplementary Material, further inquiries can be directed to the corresponding authors.
